# Men Traveling Away from Home Are More Likely to Bring Malaria into High Altitude Villages, Northwest Ethiopia

**DOI:** 10.1371/journal.pone.0095341

**Published:** 2014-04-18

**Authors:** Kassahun Alemu, Alemayehu Worku, Yemane Berhane, Abera Kumie

**Affiliations:** 1 Department of Environmental and Occupational Health and Safety, Institute of Public Health, College of Medicine and Health Sciences, University of Gondar, Gondar, Ethiopia; 2 Department of Epidemiology and Biostatistics, School of Public Health, College of Health Sciences, Addis Ababa University, Addis Ababa, Ethiopia; 3 Addis Continental Institute of Public Health, Addis Ababa, Ethiopia; 4 Department of Health Management, Environmental Health, and Behavioral Sciences, School of Public Health, College of Health Sciences, Addis Ababa University, Addis Ababa, Ethiopia; Université Pierre et Marie Curie, France

## Abstract

**Background:**

Information about malaria risk factors at high altitudes is scanty. Understanding the risk factors that determine the risk of malaria transmission at high altitude villages is important to facilitate implementing sustainable malaria control and prevention programs.

**Methods:**

An unmatched case control study was conducted among patients seeking treatment at health centers in high altitude areas. Either microscopy or rapid diagnostic tests were used to confirm the presence of *plasmodium species*. A generalized linear model was used to identify the predictors of malaria transmission in high altitude villages.

**Results:**

Males (AOR = 3.11, 95%CI: 2.28, 4.23), and those who traveled away from the home in the previous month (AOR = 2.01, 95% CI: 1.56, 2.58) were strongly associated with presence of malaria in high altitude villages. Other significant factors, including agriculture in occupation (AOR = 1.41, 95% CI: 1.05, 1.93), plants used for fencing (AOR = 1.70, 95% CI: 1.18, 2.52) and forests near the house (AOR = 1.60, 95% CI: 1.15, 2.47), were found predictors for malaria in high altitude villages.

**Conclusion:**

Travel outside of their home was an important risk of malaria infections acquisition. Targeting males who frequently travel to malarious areas can reduce malaria transmission risks in high altitude areas.

## Introduction

Malaria has unstable transmission patterns in high land areas of Ethiopia, and its occurrence is characterized by frequent and often large-scale epidemics due to the low immunity level of highland populations. Most of the malaria infections are due to *plasmodium falciparum* and *plasmodium vivax* [*1*].

Malaria transmission is highly variable and the acquisition risk for individuals is unevenly distributed even within a neighborhood in highland areas. A number of studies have been conducted to understand malaria risk factors at altitudes, less than 2000 meters above sea level [2]–[6] and more than 2000 meters above sea level in central Ethiopia. However, malaria transmission varies in space and time [7] as the country has diverse topography and climate [8], so information about the transmission of malaria risk factors in high altitudes in northwest Ethiopia is limited [9].

In Ethiopia, malaria intervention strategies have been targeted to areas located at less than 2000 m above sea level [10], [11], and intervention efforts in highland areas are limited. The exclusion of these areas in unstable transmission zones can limit the success of the overall malaria prevention and control activities. Therefore, understanding the factors which determine the risk of malaria transmission can facilitate implementing sustainable malaria control and prevention intervention. The aim of this study was to identify the risk factors that drive malaria transmission in high altitude villages in northwest Ethiopia.

## Methods

### Study Area and Settings

The study was conducted in Dabat district, northwest Ethiopia between August 2012 and May 2013. The district is about 1187.93 square km and has about 160 thousand population [12]. The district is generally hilly and the altitude is estimated to range from 1500 m to 3000 m above sea level, divided into three highland fringes zones. The high transmission and epidemic prone (between1500 and 1750 m), highland fringes with low transmission and epidemic prone (between 1750 and between 2000 m), and highlands affected by occasional epidemics (between 2000 and 2500 m) [1]. Agriculture is the main occupation of the residents. The study included the peak of the malaria season (between September and November). The annual rainfall was 740 mm in 2011 and 481 mm in 2012 [13]. Malaria transmission is seasonal and *Plasmodium falciparum* is the dominant species. The district has four health centres and twenty-nine health posts which provide health services for the people in the district.

### Study Design and Study Population

A health facility-based, unmatched case-control study was conducted. Patients who are fifteen years and above, who had history of fever in the previous 72 hours, visited one of the health centers for health seeking, and permanent residents in the Dabat district were included in the study. Patients who were extremely ill and had taken malaria treatment in the previous 30 days were excluded from the study.

### Sample Size Determination

The sample size was calculated using the Stat Calc module of Epi Info for windows [14] software with an assumption of 95% level of confidence, 80% power, odds ratio of 1.64, a 1∶2 case to control ratio, and assuming that 7.5% of the controls had travelled overnight in the past month [15]. The planned total sample size at the beginning of the study was 1767 (1178 controls and 589 cases). During the actual data collection period, 613 cases (positive thick film or RDT for any malaria parasite species) and 842 controls (negative thick film or RDT for all malaria species), a total of 1455 patients, were able to include in the study. The power for the actual sample size included in the analysis was calculated and found to be 78.8%, which is almost the same as the power assumed at the beginning of the study. Malaria testes were carried out at the health centres and patients were selected and classified into cases and controls. Cases were persons presenting at the four health facilities with a positive thick blood smear laboratory test or RDT and microscopy together. Controls were persons presenting at the same four health facilities whose thick blood smear tests or RDT and microscopy together results were negative for malaria infection.

### Data Collection

Microscopy and RDT were used to confirm the presence of *Plasmodium* species on the tested patients (confirmed malaria and no confirmed malaria). Microscopy is the standard method to diagnose malaria. RDTs offer the possibility to extend accurate malaria diagnosis [16]. Malaria infections were defined as the presence of *Plasmodium* parasites in the blood, confirmed by the presence of parasites in peripheral blood by microscopy, or malaria antigenaemia by using RDT and microscopy together [17].

A pretested and structured questionnaire was used to collect individual information. The study tool which comprised of socioeconomic characteristics, malaria preventive measures such as insecticide treated nets (ITNs) use, indoor residual spray (IRS), history of travel in the last month preceding the interview, ITN utilization during travel, travel place, and the homestead surroundings. All tested slides were stored at each health facility and the samples of ten percent of the patients were collected randomly for further quality control activities. The questionnaire was prepared in English and translated into the Amharic language (the local as well as national language) and translated back to English by independent language experts.Using principal components analysis (PCA) [18] and based on the ownerships of specific household items a wealth index was constructed for the patients.

Data entry, cleaning and editing of data were performed using the EpiData ( http://www.epidata.dk ) software version 3.02. All patient data were cross-linked to their villages and *kebeles*. The Data were double entered using the EpiData.

### Statistical Analysis

Data analysis was done using the R statistical software version 3.0.1 [19] and STATA version 12.0 [20]. Stratification and multivariate analysis were employed to control confounders. Mantel–Haenszel test, are used to produce a single weighed estimate of exposure effect, which is adjusted for the effects of the confounding factor [21], [22]. A summary effect estimate was calculated which in contrast to the crude estimate, would take into account the confounding effect of the stratifying variable. Weighted averages of the individual stratum-specific estimates were taken by choosing a set of weights that maximizes the statistical precision of the adjusted effect estimate. The magnitude of confounding was evaluated by observing the degree of discrepancy between the crude and adjusted estimates. If there is no difference between these two estimates, the observed exposure–outcome effect was not confounded by the potential confounding variable. A large difference indicates the presence of confounding and implies that the adjusted summary measure is a better estimate of the effect of the exposure on the outcome of interest than the crude summary measure, since it removes the effect of the confounder.

The relationship between malaria risks and associated factors was analyzed using the generalized linear model (GLM). GLM was used to identify the predictors of malaria transmission in high altitude villages in which the outcome variables were measured on a binary scale i.e. presence or absence of malaria infection. It was used to relate the response variable to the predictor variables via a logit link function [23], [24]. GLM permits the adaptation of procedures for model building and model checking. The deviance was used to compare alternative models during model selection. Change in the deviance was used to measure the extent to which the fit of the model improves when additional variables were included. The model fitted in two steps to avoid confounding effects was fitted to each predictor variable one at a time. Then, the predictors with p<0.25 were retained in a multivariate logistic regression model. The standardized residuals for binomial data were checked for approximate normal distribution. For factors, a factor level with a greater coefficient indicates greater odds of malaria.

### Ethical Issues

The protocol was approved by the University of Gondar Ethical Review Committees. Written consent was sought from all study participants. For illiterate participants, the consent was read by literate ones who they could trust and thumb-printed consent was obtained thereafter. For children who participated in this study, study aims and procedures were explained to parents/guardians; their understanding was confirmed through an interview before written or thumb-printed consent was obtained. Clients were assured by the study coordinator that interviews were completely voluntary and had no any risks. Patients positive for malaria tests were given anti malaria treatment. All data were confidential and names were not linked to the data in any way.

## Results

### General profile of the study participants

A total of 613 cases (positive thick film or RDT for any malaria parasite species) and 842 controls (negative thick film or RDT for all malaria species) were participated in the study with 82.3% response rate. Of the total malaria cases, 380(61.9%) were diagnosed with microscopy and 234(38.1%) were using RDT and microscopy. The majority of the patients, 804 (55.3%), visited Dabat Health Center while 368(25.3%) went to Weken Health Center. Most of the patients, 331 (53.9%), had *Plasmodium falciparum*, the dominant *plasmodium species*, while 169(27.5%) and 114(18.6%) *plasmodium vivax* and mixed species respectively. The mean (±SD) and the median age of the participants was 26.9±10.5 years and 24 years respectively with inter quartile range of 10 years. Of the 1455 participants, the majority, 1116 (76.7%) were males; 1421 (97.7%) were Orthodox Christians, 1433 (98.5) were Amhara by ethnicity; 1184(81.4%) were agricultural workers, 1032(70.9%) were 15 to 29 years of age group, and 485(33.3) low class patients (Table 1).

**Table 1:** Characteristics of the study population in high altitude villages northwest Ethiopia, 2013.

Variables		Malaria		Total (%)
		Cases (%)	Controls (%)	
Sex	Female	64(10.4)	275(32.7)	339(23.3)
	Male	550(89.6)	566(67.3)	1116(76.7)
Age	15–29	482(78.5)	550(65.4)	1302(89.5)
	30–44	103(16.8)	195(23.2)	298(20.5)
	45–64	26(4.2)	90(10.7)	116(7.9)
	> = 65	3(0.5)	6(0.7)	9(0.6)
Religion	Muslim	7(1.1)	27(3.2)	34(2.3)
	Christian	607(98.9)	814(96.8)	1421(97.7)
Occupation	Non agriculture	86(14.0)	185(22.0)	271(18.6)
	Agriculture	528(86.0)	656(78.0)	1184(81.4)
Ethnicity	Amhara	605(98.5)	828(98.5)	1433(98.5)
	Tigre	6(1.0)	12(1.4)	18(1.2)
	Oromo	3(0.5)	1(0.1)	4(0.3)
Wealth index	Lowest	175(28.5)	310(36.9)	485(33.3)
	Middle	214(34.9)	271(32.2)	485(33.3)
	Higher	225(36.7)	260(30.9)	485(33.3)
Own ITN	No	427(69.5)	556(66.1)	983(67.6)
	Yes	187(30.5)	285(33.9)	472(32.4)
Number of ITNs available	ne	120(19.5)	153(18.2)	273(18.8)
	Two and more	494(80.5)	688(81.8)	1182(81.2)
Slept in ITN yesterday night	No	43(23.0)	95(33.3)	138(29.2)
	Yes	144(77.0)	190(66.7)	334(70.8)
IRS sprayed home	No	586(95.4)	798(94.9)	1387(95.3)
	Yes	28(4.6)	43(5.1)	68(4.7)
Travel away from home	No	306(49.8)	614(73.0)	920(63.2)
	Yes	308(50.2)	227(27.0)	535(36.8)
Travel away home frequency	< = 3 times	41(6.7)	28(33.3)	69(4.7)
	> = 3 times	573(93.3)	813(96.7)	1386(95.3)
Usage of ITN during travel	No	224(72.7)	181(79.7)	405(75.7)
	Yes	84(27.3)	46(20.3)	130(24.3)
Months travel most	July to August	183(57.7)	82(26.3)	265(48.3)
	September to November	83(26.2)	183(58.7)	186(33.9)
	December to February	20(6.3)	26(8.3)	46(8.4)
	March to May	31(9.8)	21(6.7)	52(9.5)

Of all the study patients, 472 (32.4%), owned at least one ITN, 199 (42.2%) of whom had two and more ITNs; 273 (57.8%) had only one ITN each. Three hundred thirty-four, (70.8%) of the patients reported to sleep under ITN in the previous night. Sixty- eight (4.7.0%) of the patients reported that their houses were sprayed with IRS (Table 1).

Out of the patients who visited health centers for medical care, 535 (36.8%) traveled away from their permanent residence during the last month prior to their visit to health institutions. Of these who travelled outside of their permanent residence, 308 (50.2%) were positive for plasmodium parasites. Most of them, 396 (73.6%) travelled to areas such as Abrahjira, Abderafi, Metema, Quara and Dansha where malaria transmissions are endemic. Those who were engaged in agriculture, 1184 (81.4%) travelled most. The main purpose of travel for 373 (69.7%) of the patients were looking for temporary jobs. One hundred thirty- five (24.3) of them slept under ITN during their travel (Table 2). Of the travelers, 383 (71.6%) were from high altitudes villages ((Table 3). Two hundred sixty five (48.3%) of the patients travelled most from July to August, and 186 (33.9%) from September to November, that is, during cultivation and harvesting seasons. Most of the travelers 1032 (70.9%) were between 15 and 29 years aged. Of these, 403 (75.3%) were positive for *plasmodium parasites*, and 495 (92.5%) were males (Table 2).

**Table 2:** Characteristics of homestead environment and travel history in high altitude villages of northwest Ethiopia, 2013.

Variables		Malaria		Total (%)
		Cases (%)	Control (%)	
Stagnant water	No	513(83.6)	718(85.4)	1231(84.6)
	Yes	101(16.4)	123(14.6)	224(15.4)
Swampy	No	486(79.2)	646(76.8)	1132(77.8)
	Yes	128(20.8)	195(23.2)	323(22.2)
Bore hole	No	566(92.2)	796(94.6)	1362(93.6)
	Yes	48(7.8)	45(5.4)	93(6.4)
Flowers	No	595(96.9)	823(97.9)	1418(97.5)
	Yes	19(3.1)	18(2.1)	37(2.5)
Plants used for fencing	No	535(87.1)	782(93.0)	1317(90.5)
	Yes	79(12.9)	59(7.0)	138(9.5)
Long grass surroundings	No	516(84.0)	710(84.4)	1226(84.3)
	Yes	98(16.0)	131(15.6)	22915.7)
Short grass in surroundings	No	439(71.5)	639(76.0)	1078(74.1)
	Yes	175(28.5)	202(24.0)	377(25.9)
Bushes	No	440(71.7)	563(66.9)	1003(68.9)
	Yes	174(27.3)	278(33.1)	452(31.1)
Forest	No	100(16.3)	270(32.1)	370(25.4)
	Yes	514(83.7)	571(67.9)	1085(74.6)
Altitude of the village	1500–1750 m	88(14.3)	125(14.9)	213(14.6)
	1751–2000 m	62(10.1)	82(9.8)	144(9.9)
	Above 2000 m	464(75.6)	634(75.3)	1098(75.5)

**Table 3:** Characteristics of malaria risk factors by altitude in highland villages, northwest Ethiopia, 2013.

Variables		Altitude		
		1500–1750 m	1750–2000 m	Above 2000 m
Sex	Female	47(22.1%)	25(17.4%)	267(24.3%)
	Male	166(77.9)	119(82.6%)	831(75.7%)
Age	15–29	159(74.6%)	88(61.1%)	785(71.5%)
	30–44	38(17.8%)	37(25.7%)	223(20.3%)
	45–64	16(7.6%)	17(11.8%)	83(75.6%)
	> = 65		2(1.4%)	7(0.64%)
Travel away home	No	120(56.3%)	85(59.0%)	715(65.1%)
	yes	93(43.7%)	59(41.0%)	383(34.9%)
ITN own	No	24(11.3%)	11(7.6%)	948(86.3%)
	Yes	189(88.7%)	133(92.4%)	150(13.7%)
IRS sprayed	No	164(77%)	130(90.3%)	1093(99.5%)
	Yes	49(23%)	14(9.7%)	5(0.5%)
Plants used for fence	No	160(75.1%)	140(97.2%)	1017(92.8%)
	Yes	53(24.9%)	4(2.8%)	80(7.2%)
Forest near homestead	No	47(22.1%)	36(25.0%)	287(26.1%)
	Yes	166(77.9%)	108(75.0%)	811(73.9%)
Stagnant water near the house	No	172(80.8%)	97(67.4%)	962(87.7%)
	Yes	41(19.2%)	47(32.6%)	135(12.3%)
Borehole near the house	No	197(92.5%)	136(94.4%)	1029(93.7%)
	Yes	16(7.5%)	8(5.6%)	69(6.3%)
Malaria infection	No	125(58.7%)	82(56.9%)	634(57.7%)
	Yes	88(41.3%)	62(43.1%)	464(42.3%)
Malaria species	*P.falciparum*	54(61.4%)	38(61.3%)	239(51.6%)
	*P.vivax*	12(13.6%)	7(11.3%)	94(20.3%)
	mixed	22(25.0%)	17(27.4%)	130(28.1%)

The micro-ecological environment was assessed with regard to malaria transmission, and it was found out that types of trees used for fences around homestead were 138 (9.5%). One thousand ninety eight, (75.5) of the patients resided at an altitude above 2000 m (Table 2). The majority, age group between 15 and 29, 738(76.1%), malaria patients 464(75.6%), males 831(74.5), *p. falciparum species* 237(72.2%) were from high altitudes villages (Table 3).

### Stratification analysis for checking confounders and effect modifiers

The stratification analysis using Mantel-Haenszl method showed that the discrepancy between the crude and adjusted estimates had not much confounded across all risk factors. Though a large differences not seen the *adjusted* summary measure is a better estimate of the effect of the exposure on the outcome of interest than the *crude* summary measure, since it removes the effect of the confounder. Male (OR_MH_ = 4.08; 95%CI: 3.00, 5.40), and travel away home (OR_MH_ = 1.966; 95%CI: 1.59, 2.43) were among risk factors for malaria transmission. Moreover, the strata did not show opposing measure of association. There was no evidence of effect modification (Table 4).

**Table 4:** Stratification analysis of malaria risk factors stratifying by altitude uses the Mantel Haenszel method in highland villages, northwest Ethiopia, 2013.

Variables	COR	1500–1750 m	1750–2000 m	Above 2000 m	Adjusted (Mantel-Haenszel)			
		OR	OR	OR	OR_MH_	95%CI	X^2^	P value
Sex	4.07	3.83	1.13	4.86	4.08	3.00,5.40	95.6	0.0001
Occupation	1.76	4.0	1.54	1.66	1.78	1.33,2.33	16.1	0.0001
ITN own	1.015	1.19	0.87	1.05	1.057	0.77,1.44	0.1	0.7
IRS sprayed	0.644	0.62	0.51	0.91	0.616	0.35,1.08	2.9	0.08
Travel away home	1.969	1.02	2.68	2.15	1.966	1.59,2.43	39.8	0.0001
Plants used for fence	1.94	3.15	1.37	1.65	2.02	1.41,2.91	14.8	0.0001
Long grass near the house	1.011	1.52	0.79	0.90	1.208	0.76,1.38	0.02	0.89
Forest near the house	2.379	1.48	2.32	2.60	2.379	1.84,3.08	44.8	0.0001
Short grasses near the house	1.247	1.60	0.67	1.33	1.265	0.99,1.61	3.7	0.055
Bushes	1.55	1.47	1.04	0.66	1.549	0.80,2.99	3.13	0.188

OR_MH_: adjusted odds ratio using Mantel-Haenszel method, COR: crude odds ratio.

### Multivariate model using generalized linear model

The generalized linear model identified that males had 3.1 times higher risk for malaria infection compared with females (AOR = 3.1; 95% CI: 2.34, 4.23, p<0.0001). Patients mainly engaged in agricultural activities were at a higher risk for malaria infection by 40% compared with their non-agricultural counter parts (AOR = 1.40; 95% CI: 1.05, 1.91, p<0.02). Furthermore, compared to patients who did not travel away from their permanent residence in the month immediately prior to the study, those who traveled had about 2.01 times higher risk for malaria infection (AOR = 2.01, 95% CI: 1.56, 2.49, p value <0.0001). Patients who used trees for fences of their homestead were more likely to contract malaria compared to those who did not have such fences (AOR = 1.71; 95% CI: 1.19, 2.47, p<0.003). The religion of the patient, age groups, presence of short and long grass, swampy, stagnant water, dig holes, broken clays, discarded tins and equipment around homesteads had not appeared to have a significant impact on the probability of acquiring malaria infection (Table 5).

**Table 5:** Predictors of malaria transmission risk in high altitude villages of northwest Ethiopia, 2013.

Parameters		Beta Coefficients	COR	P value	Beta Coefficients	AOR	Wald 95% Confidence Limits	
							Lower	Upper
Sex	Female		1			1		
	Male	1.43	4.2	0.001	1.15	3.11	2.28	4.23
Occupation	Non agriculture		1			1		
	Agriculture	0.55	1.73	0.001	0.36	1.41	1.05	1.93
Travel away from home	No		1			1		
	Yes	1.01	2.75	0.001	0.68	2.01	1.58	2.56
Plants used for fencing	No		1			1		
	Yes	0.66	1.9	0.001	0.54	1.7	1.18	2.52
Forests near house	No		1			1		
	Yes	0.89	2.4	0.001	0.49	1.6	1.15	2.47
Borehole near house	No		1			1		
	Yes	0.41	1.5	0.06	0.35	1.42	0.88	2.18
ITNs available	No		1			1		
	Yes	−0.16	0.9	0.17	−0.18	0.83	0.66	1.06

COR: crude odds ratio, AOR: adjusted odds ratio.

## Discussion

This study characterized risk factors for malaria in high altitude villages in northwest Ethiopia. Males, history of travel in the month preceding the interview, plants used for fencing, presence of forests near house and agricultural occupation were found statistically significant predictors for malaria risks.

In this study, malaria transmission was found to vary between males and females. The high malaria risk observed in males as compared with females might be due to different exposure or other behavioral risk factors. Females are more likely to stay at home and travel less, and may use insecticide treated nets appropriately[5]. Males may be at risk due to larger travel from home for harvesting, seasonal job seeking and other social affairs [25].

Patients who traveled to malaria endemic areas prior to the survey were more likely to the risk of contracting malaria compared with those who did not travel. This finding is consistent with those of other studies done elsewhere in Ethiopia and Kenya [25], [26]. This may be due to the movement of non immune people from higher malaria free altitudes to areas such as Humara, Metema, Quara, Dansha, Sanja, and Abrah Jira which are lower-laying malaria endemic areas leading to severe infections in people who cannot take preventative measures as they are away from home. Non-immune individuals are predominantly susceptible to malaria if exposed to an infected mosquito, particularly in areas with high levels of transmission [27]. The movements, in search of temporary jobs and other social affairs are predominantly seasonal and take place when malaria transmission is ongoing. By doing so, they may be exposed to foci of vector activity in these areas. A seasonal movement has been associated with epidemics in Kenya [28]. Hence, travel away from home is an important predictor of malaria transmission in high altitude villages, northwest Ethiopia. However, that almost half of the malaria infected patients did not travel to malaria risk areas may imply some level of high altitude village transmission or that there might be regular travels which are not reported. Hence, the true cause for the transmission of malaria among people who live in high altitudes and do not travel needs to found out.

In this study, malaria interventions including ownership of ITN, sleeping in ITN on the previous night, number of ITNs available in the household, and spraying of houses with IRS had not brought any impact on malaria transmission though it is generally accepted that they reduce malaria infection and mortality. The findings are consistent with others reported that ownership or use of an ITN showed lack of protective effect against malaria in the country [4], [25], [29]. However, spraying IRS on the walls of houses reduced the risk of malaria [3], [29], [30]. The possible explanation for these findings may be that patients underreported possession, or the ITNs may be old and incompetent to protect or those who use bed nets at home fail to do so when traveling, or there are no adequate numbers of individuals possessing ITN as the major malaria interventions have given priority to malarious areas less than 2000 m [11].

The study focused on the passive case detection which may be limited to show the true pictures of malaria risk factors in high altitude villages. Respondents might not tell the facts as the interview took place at health centers and did not do physical observation of the housing conditions, homestead environment, possession and utilization of ITNs. Though questions were amended and indicated to know the months and seasons people travelled, travel histories of patients were assessed retrospectively and may reflect recall bias in accurately reporting travel patterns. The cases and controls were selected from health institutions which might introduce selection bias as patients often do not represent the general population, since the enrollments of the patients were based on the outcome of interest. More males than females are selected in the study. This can cause an overestimate or underestimate of the association and the population studied not accurately reflects the target population. Careful consideration was taken during data collection and analysis stages to minimize the bias. The data were analyzed using stratified and multivariate analysis for controlling confounders and assess effect modifiers. The unmeasured confounders are not controlled but the effect on the result is minimal.

This finding would suggest that understanding the socio-economic and behavioral characteristics of the travelers, type of ecological setting, and availability of malaria vectors in the villages, timing and type of people who travel and considering environmental risk factors in high altitude villages are indispensable for successful and sustainable malaria control measures.

## Conclusion

These results provide further insights for identifying the risk of malaria transmission in high altitudes villages. Travel away from home is an important predictor of malaria transmission in high altitude villages where vulnerable individuals travel through malaria endemic areas. Therefore, these results imply that malaria intervention approaches are significant and should be designed particularly for non-immune individuals who reside in high altitudes many of whom frequently travel to malarious areas.

## Acknowledgments

We are grateful to Dabat Health Department's commitment to facilitate the research activities. We also thank the Ethiopian Central Statistics Agency for providing shape files for the study areas. We also appreciate the University of Gondar Research Center (Dabat Demographic surveillance site) for providing GPS and trained data collectors. We forward our appreciation to data collectors and study participants and supervisors.
